# Protoporphyrin IX delayed fluorescence imaging: a modality for hypoxia-based surgical guidance

**DOI:** 10.1117/1.JBO.27.10.106005

**Published:** 2022-10-10

**Authors:** Arthur F. Petusseau, Petr Bruza, Brian W. Pogue

**Affiliations:** a Dartmouth College, Thayer School of Engineering and Dartmouth Cancer Center, Hanover, New Hampshire, United States; b University of Wisconsin–Madison, Department of Medical Physics, Madison, Wisconsin, United States

**Keywords:** fluorescence-guided surgery, hypoxia, protoporphyrin IX, time-gated imaging, molecular imaging

## Abstract

**Significance:**

Hypoxia imaging for surgical guidance has never been possible, yet it is well known that most tumors have microregional chronic and/or cycling hypoxia present as well as chaotic blood flow. The ability to image oxygen partial pressure (${\mathrm{pO}}_{2}$) is therefore a unique control of tissue metabolism and can be used in a range of disease applications to understand the complex biochemistry of oxygen supply and consumption.

**Aim:**

Delayed fluorescence (DF) from the endogenous molecule protoporphyrin IX (PpIX) has been shown to be a truly unique reporter of the local oxygen partial pressure in tissue. PpIX is endogenously synthesized by mitochondria in most tissues, and the particular property of DF emission is directly related to low microenvironmental oxygen concentration. Here, it is shown that PpIX has a unique emission in hypoxic tumor tissue regions, which is measured as a DF signal in the red to near-infrared spectrum.

**Approach:**

A time-gated imaging system was used for PpIX DF for wide field direct mapping of ${\mathrm{pO}}_{2}$ changes. Acquiring both prompt and DF in a rapid sequential cycle allowed for imaging oxygenation in a way that was insensitive to the PpIX concentration. By choosing adequate parameters, the video rate acquisition of ${\mathrm{pO}}_{2}$ images could be achieved, providing real-time tissue metabolic information.

**Results:**

In this report, we show the first demonstration of imaging hypoxia signals from PpIX in a pancreatic cancer model, exhibiting >5X contrast relative to surrounding normal oxygenated tissues. Additionally, tissue palpation amplifies the signal and provides intuitive temporal contrast based upon neoangiogenic blood flow differences.

**Conclusions:**

PpIX DF provides a mechanism for tumor contrast that could easily be translated to human use as an intrinsic contrast mechanism for oncologic surgical guidance.

## 1 Introduction

Surgical guidance with fluorescent tracers has seen significant adoption in the last decade,^1^ including the ability to image tissue perfusion.^2^^,^^3^ More recently, tissue metabolism tracers have been approved for human use, such as protoporphyrin IX (PpIX),^4^^,^^5^ which is induced by oral or topical administration of 5-aminolevulinic acid (ALA).^6^ One of the most striking things about the ALA-PpIX system is that it is produced throughout most tissues of the body and demarcates glioma tumors in neurosurgery, skin lesions, and bladder cancers. However, a less studied PpIX phenomenon in surgical guidance is its delayed fluorescence (DF) signal, which is directly related to the ambient oxygen level around it.^7^^,^^8^ The lack of oxygen, or hypoxia, induces a strong DF component that has significant relevance to oncology, where the vast majority of tumors have microregional hypoxia present.^9^

Optical probes based on the quenching of their triplet state by oxygen have been used in the past^10^ to image oxygen distribution in tumors.^11^^,^^12^ However, real-time mapping of oxygen was never achieved before. The molecule PpIX produces a DF signal that is a direct result of low oxygenation because there is reverse intersystem crossing from the triplet state of the molecule. Since the signal comes from the triplet state, which is normally quenched by molecular oxygen, its intensity is a direct reporter of the lack of oxygen in tissue. Additionally, since the molecule PpIX is an endogenously produced species from the administration of ALA, it is well tolerated and widely available in a number of pro-drug formulations.

Previous work showed that oxygen partial pressure (${\mathrm{pO}}_{2}$) levels could be estimated from measurement of the PpIX delayed emission lifetime,^8^^,^^13^ using a fit to the Stern–Volmer equation. One great advantage of this approach is that lifetime is a quantitative measurement independent of PpIX concentration and tissue optical properties. Because of how weak the DF signal is relative to the prompt fluorescence (PF), single photon cameras have been needed to image it and reconstruct spatial ${\mathrm{pO}}_{2}$ distributions. Time-resolved data are acquired using temporal gates with an increasing time delay relative to a laser excitation pulse. For robust reconstruction of the DF lifetime, a minimum of six gates have been needed,^13^ bringing the total signal integration time longer. Thus, this approach to imaging ${\mathrm{pO}}_{2}$ values results in a slow frame rate because of the limited signal per time bin.

Here, we propose a time-gated optical system capable of real-time imaging of hypoxia, relying on a simpler ratiometric approach to imaging the hypoxia signal. To optimize signal gathering, frames were exposed over the whole DF emission decay time. DF was then normalized by the PF signal to factor out variations due to changes in PpIX concentration or tissue absorption.

## 2 Materials and Methods

### 2.1 Ethics Statement

Experimental procedures involving live animals were carried out in accordance with the protocols approved by Dartmouth Institutional Animal Care and Use Committee (Protocol Number 00002059). Subcutaneous and intraperitoneal injections were performed under anesthesia that was induced and maintained with isoflurane. All efforts were made to minimize animal suffering.

### 2.2 Tumor Cell Lines

The human pancreatic cancer cell line BxPC3 was purchased from ATCC. Cells were grown in culture media in a humidified incubator at 37°C and 5% ${\mathrm{CO}}_{2}$ in RPMI with 10% (v/v) fetal bovine serum (FBS), 100  U/mL penicillin, and 100  μg/mL streptomycin. When ready for use, the cells were trypsinized, counted, pelleted, and resuspended in Matrigel for injection.

### 2.3 Animal Preparation

All procedures followed the protocol approved by the Dartmouth Institutional Animal Care and Use Committee. Nude female mice 6 to 8 weeks of age (Charles River Labs, Wilmington, Massachusetts) were involved in this study. The mice were housed in the Dartmouth central animal facility and fed a special diet —MP biomedical purified diet. The mice were inoculated with a single subcutaneous injection of $10^{6}$ human pancreatic adenocarcinoma BxPC3 cells, under the skin on the flank. After ∼3 weeks of growth, animals were chosen for imaging when their tumor diameter reached ∼8  mm in size. On the day of the initial use, mice were anesthetized, and ALA was either i.p. injected (250  mg/kg) 6 h prior to imaging or topically applied onto the region of interest 3 h before imaging as Ameluz, an ointment containing 10% ALA (Biofrontera AG, Germany). Note that the dose of five-ALA delivered to mice is high compared to the dose usually administered to patients (20  mg/kg b.w. orally in the neurosurgical context). This difference is because dosing ranges from mice to humans are based upon $\mathrm{mg}/m^{2}$ estimate values, and the estimated human equivalent dose is about 12.3× lower than the mouse dose.^14^

### 2.4 Camera and Setup

**Fig. 1:**
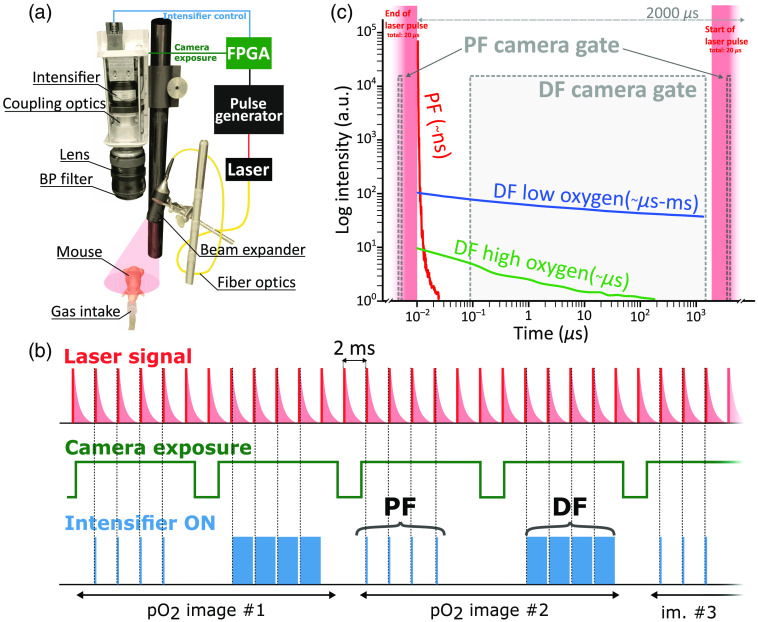
(a) Schematic of the PpIX DF acquisition setup with the block diagram of the camera intensifier external control. (b) Time diagram of the sequential acquisition of PF and DF showing correspondence between laser excitation, camera exposure, and intensifier control. The number of gates per image is illustrative and can be varied depending on fluorescent signal intensity. (c) Intensity and time decay of PpIX PF and DF are shown at high and low oxygen levels in a double log scale. Camera acquisition gates are overlayed to show the timing of the sequential acquisition. BP = bandpass, DF = delayed fluorescence, PF = prompt fluorescence. Note that the logarithmic scale only applies to the fluorescent decays and that the laser excitation pulse width is 20  μs.

The images were acquired using a highly sensitive intensified CMOS camera (C-Dose, DoseOptics LLC, New Hampshire) synchronized with a 50-mW average output power 635 nm modulated diode laser (BWT, China), used with 20-μs pulses at a repetition rate of 500 Hz. The laser was partially collimated to irradiate an area with 8-cm diameter, leading to a temporally averaged irradiance of $500\;\mu W/{\mathrm{cm}}^{2}$ at the sample. Irradiance was measured using a power meter (PM100D, Thorlabs, New Jersey) coupled with a photodiode power sensor (S121C, Thorlabs, New Jersey). The intensifier was directly controlled by an external FPGA (XEM7360, Opal Kelly, San Jose, California) [Fig. 1(a)], which was synchronized to the laser pulses and allowed custom pulse sequencing and control of the image acquisition timing. As mentioned-above, the PF and DF pulses used different pulse timings driving the intensifier: for PF we used 100 ns pulse width and 10  μs delay with respect to laser pulse rising edge, whereas the DF pulses had pulse width of 1975  μs and delay of 2  μs after the laser pulse. A signal corresponding to the camera’s exposure times was used as an input to the FPGA sequencer to control the 50 laser pulse gates integrated in each image frame [Fig. 1(b)], and switch between PF and DF pulse settings for even and odd frames, respectively. Thus, PF and DF of PpIX were acquired in a sequential way, with an effective frame rate of 10 fps, allowing “real-time” reconstruction of the normalized hypoxia image (i.e., DF/PF). The fluorescent signal was passed through a 697±37  nm, OD six band-pass filter (87–771, Edmund optics, New Jersey), removing any remaining laser emission interfering with the detection spectral window. All images were acquired with 2×2  pixel binning, yielding a final image size of 800×600  pixels.

### 2.5 Image Analysis and Statistics

All images were background subtracted and median filtered spatially to remove hot pixels and readout noise. Time plots were smoothed using a 10-element sliding mean filter. Fluorescence images were overlayed in a semitransparent way over their corresponding white light images, using custom-made colormaps. Reoxygenation rate constant maps in Fig. 3(b) were calculated by per 5×5 binned pixel fitting temporal image stacks of DF images with a single exponential decay function and least squares regression method, in MATLAB. The fit was done on background subtracted image using the equation:

$$\text{Fit}=Ie^{-\tau .\text{time}},$$ (1)

where I and τ corresponded to the fitted parameters and τ is the rate constant displayed in Fig. 3(b). All box plots were plotted using a MATLAB built-in function, where the central red mark indicates the median, and the bottom and top edges of the box indicate the 25th and 75th percentiles, respectively. The whiskers indicate the most extreme data points not considered outliers. Data was gathered from the five different specimen images, by drawing 10 20×20 binned pixel ROIs distributed on the tumor surface, as well as 10 60×60 binned pixel ROIs distributed on the normal tissue surface. Contrast-to-variation ratio^15^ (CVR) was defined as

$$\mathrm{CVR}=\frac{\mu_{t}-\mu_{n}}{\sqrt{\sigma_{t}^{2}+\sigma_{n}^{2}}},$$ (2)

where $\mu_{t}$ and $\mu_{n}$ are the mean fluorescence from tumor and normal tissues, respectively, and $\sigma_{t}$ and $\sigma_{n}$ are the standard deviation of fluorescence values in tumor and normal tissue, respectively.

## 3 Results

### 3.1 Real-time Imaging of Oxygen Distribution

To evaluate the distribution of oxygen *in vivo*, PpIX was imaged using a fast intensified camera. Both PF and DF of PpIX were acquired in a sequential way [Fig. 1(b)] to obtain metabolic information of the tissue. The combination of high sensitivity and precise acquisition timing allowed one to image PpIX DF in real-time, at frame rates up to 10 frames per second (fps) (or 5 fps for the ratio R=DF/PF) despite extremely weak signal. Because the PF signal shows intensities that are orders of magnitude higher than DF [Fig. 1(c)], acquiring both signals at the same time is challenging. The intensity difference is due to the low triplet quantum efficiency of PpIX^16^ as well as the acquisition timing for both fluorescence signals. To overcome that problem, different gate widths were used for PF and DF: PF was acquired for a very short period of time (100 ns) during the excitation pulse, whereas the DF was acquired after the end of the PF decay, during a 2-ms window [Fig. 1(c)]. This temporal sampling approach allows separation of the DF and PF signals, and acquisition of them in the same camera, even though they have orders of magnitude different intensity levels. Additionally, both signals have perfectly matching spectra and instrument responses since they are the same emission band. DF signal intensity is inversely related to intracellular oxygen levels. Therefore, imaging PpIX DF allows one to identify low oxygenated areas such as tumors. The ability to recover oxygen metabolism in real time is of crucial importance for live monitoring of tumor resection during surgery.

### 3.2 *In Vivo* pO2 Dependance of PpIX DF

**Fig. 2:**
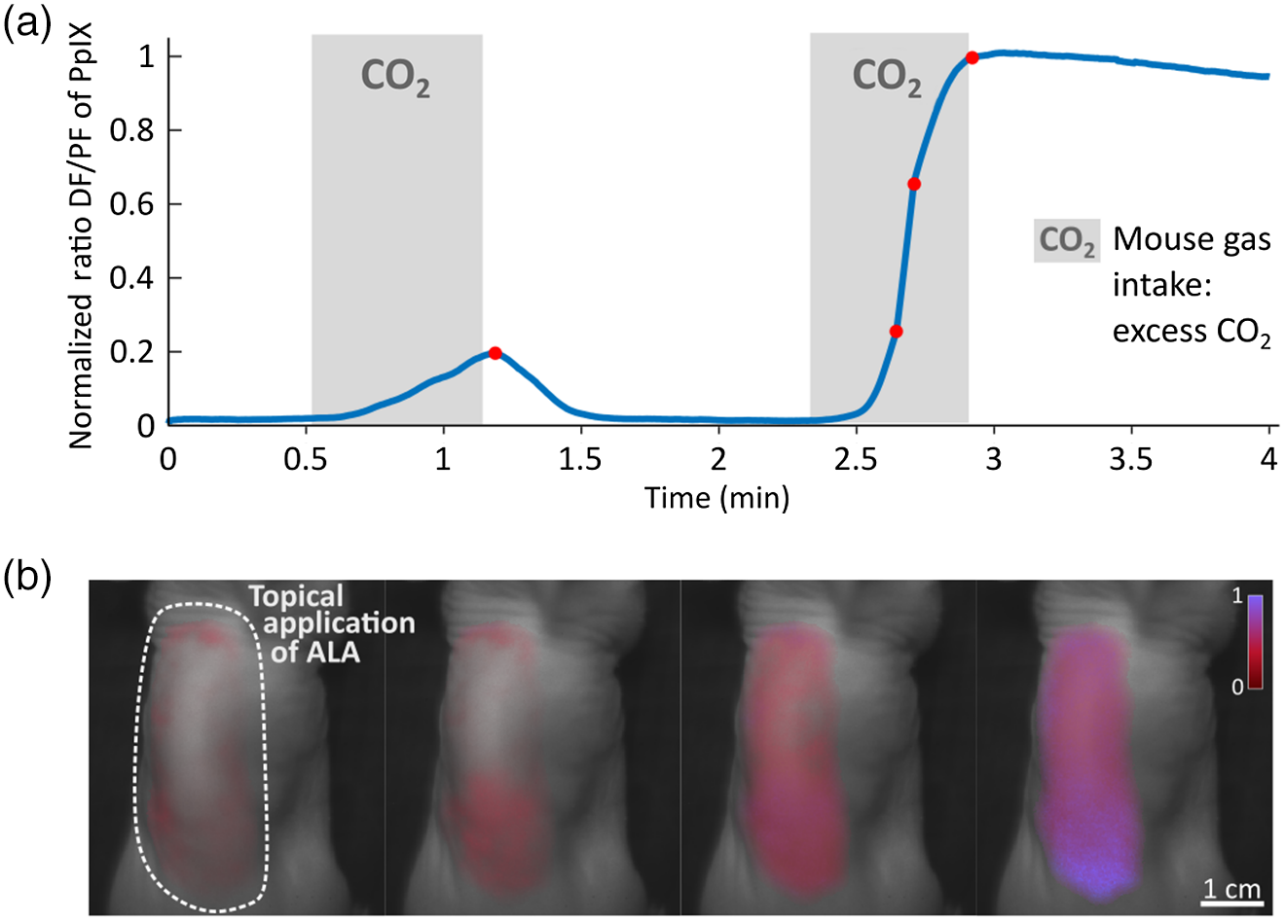
(a) Oxygen transient in a mouse model during delivery of ${\mathrm{CO}}_{2}$, implying hypoxia in tissue. ${\mathrm{CO}}_{2}$ delivery episodes correspond to an increase of the ratio DF/PF signal implying that it is inversely related to the local ${\mathrm{pO}}_{2}$. (b) Corresponding DF maps of the mouse as ${\mathrm{CO}}_{2}$ gets delivered. The increase of fluorescent signal illustrates the depletion of oxygen. Images from left to right correspond to time points represented by the red dots in (a), in the same order.

*In vivo* experiments followed the procedures described in an IACUC approved animal protocol. To demonstrate the ability of the system to measure oxygen variation *in vivo*, initial experiments used ALA applied topically as an ointment (Ameluz, Biofrontera AG, Germany) on a $∼4\;{\mathrm{cm}}^{2}$ area, on the back of a nude mouse. Pulses of ${\mathrm{CO}}_{2}$ were delivered in excess to the anesthetized mouse to provoke rapid hypoxia between periods of normal oxygen breathing. The PpIX signal variations were measured and as shown in Fig. 2(a), indicated an inverse relation between PPIX DF and ${\mathrm{pO}}_{2}$ *in vivo*. As part of this initial calibration work, both the PF and the DF signals were measured. The PF showed minimal dependency on ambient oxygen. Therefore, we considered that the measurement of the ratio R=DF/PF could allow for correction for PpIX concentration differences across tissues and for tissue pigmentation, without any errors induced due to baseline offset or wavelength filtering differences. Since the variations in PF intensity with oxygen levels are minimal compared to DF changes, PF is considered constant compared to DF. As animals were sacrificed, PF intensity was measured to drop by 5% on average, versus 90% in the case of DF, for similar initial normoxic intensities. Even though this approach provides nonabsolute measurements, it allows for the ability to monitor oxygen transients and spatial distributions in tissue. From the flat low portion of the curve shown in Fig. 2(a), it seems like the signal is reaching a low plateau at higher oxygen levels. The system can be tuned to shift the dynamic range toward higher ${\mathrm{pO}}_{2}$ values but could not cover the full range of *in vivo* ${\mathrm{pO}}_{2}$. Here, the imaging system was optimized for increased sensitivity to low oxygen levels. The fluorescent images were acquired using the system described in the previous section and illustrated in Fig. 1(a). PpIX distribution (corresponding to PF distribution) matched precisely with the skin area where ALA was applied [Fig. 2(b)]. DF maps at four different time points [corresponding to the red dots in Fig. 2(a)] are shown in Fig. 2(b). Note that for both ${\mathrm{CO}}_{2}$ delivery with identical times, the response in oxygen variation is not the same. This is mostly because the ${\mathrm{CO}}_{2}$ flows were different for both events. However, experiments have shown that the response to ${\mathrm{CO}}_{2}$ excitation can be significantly different depending on the anesthesia level, body temperature, and how much ${\mathrm{CO}}_{2}$ was delivered prior to this point.

### 3.3 Tissue Palpation Inducing Sustained Transient Hypoxia Contrast

**Fig. 3:**
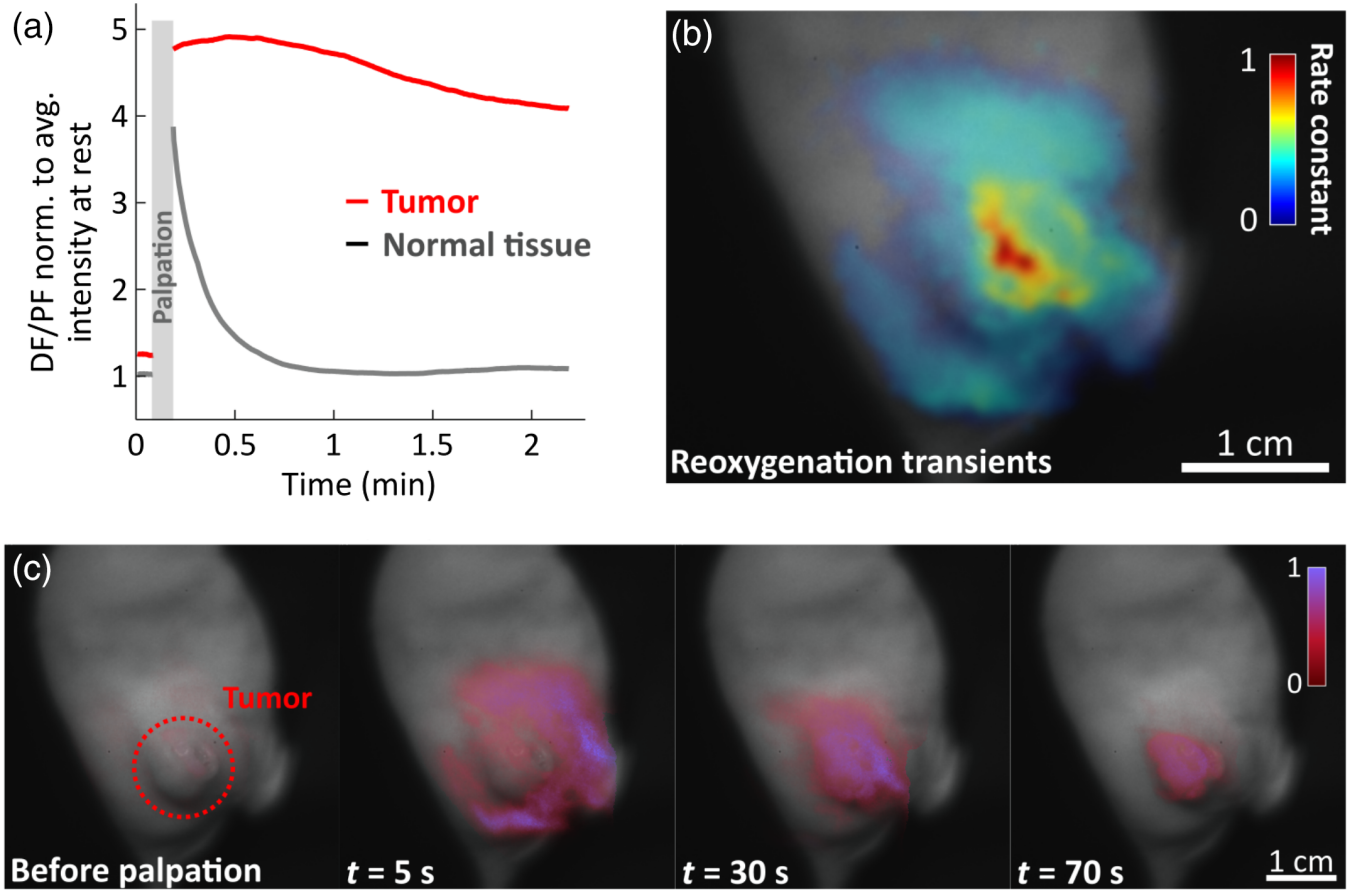
(a) Kinetics of oxygen levels in tumor tissue vs normal tissue in a murine model as tissue reoxygenate after palpation. (b) Corresponding reoxygenation time transients can be parametrized for each pixel to illustrate the kinetics of oxygen diffusion after applied pressure. Oxygen rediffusion lifetime can be fitted and plotted, showing high values for long-lasting hypoxia areas and low values fast reoxygenating areas. (c) Four snapshots of the DF map before and after palpation, as tissue reoxygenate, the remaining signal in the tumor shows that it reoxygenates slower, offering great contrast compared to normal tissue.

Most tumors have microregional chronic and/or cycling hypoxia present. Pancreatic tumors specifically, because they are quite avascular and stromal in nature, present pre-existing hypoxia, such as that described here.^17^^,^^18^ Because of their structure, pancreatic tumors do not produce significant PpIX themselves, but rather PpIX is produced throughout the body and distributed by the blood. PpIX then accumulates in tumorous tissue through the enhanced permeability and retention (EPR) effect.^19^ Here, nude mice with subcutaneous human pancreatic adenocarcinoma BxPC3 tumors were used for imaging, which was performed 6 h after intraperitoneal injection of ALA (250  mg/kg).^20^ This extended time point was chosen to allow PpIX production throughout the mouse body and subsequent accumulation in the tumor. Even though two of the five specimens analyzed in this study showed sufficient contrast naturally between tumor and normal tissue, tissues were palpated before imaging to further amplify hypoxia transiently [Fig. 3(a)]. Indeed, because of their poor vascularization, tumors reoxygenate slowly, unlike normal tissue, which has high blood pressure and consistent flow. This palpation-induced deoxygenation offered excellent contrast for up to 5 min, based on our experiments, based upon the kinetic differences shown in Fig. 3(a). This illustrates the reperfusion and oxygenation kinetics in normal vs tumorous tissue before and after tissue palpation. Before applying pressure to the tissue, PpIX DF intensity levels are quite low. Once pressure is applied, as the blood is driven away, tissues deoxygenate and emit a strong DF signal as shown in Fig. 3(c). Approximately a minute after palpation, normal tissues recover their normal ${\mathrm{pO}}_{2}$, leaving the tumor with high contrast, as shown on the far-right image of Fig. 3(c). Additionally, temporal evolution of DF/PF map R can be parametrized for each pixel to display the kinetics of oxygen diffusion after palpation [Fig. 3(b)]. In such case, care shall be taken to avoid motion artifacts, which to a certain degree affected the results in Fig. 3(b). To correlate tumor type, size, and structure of the tumor with the need for palpation and corresponding pressure, further studies are needed.

### 3.4 PpIX DF Contrast in Tumors Relative to Normal Tissues

**Fig. 4:**
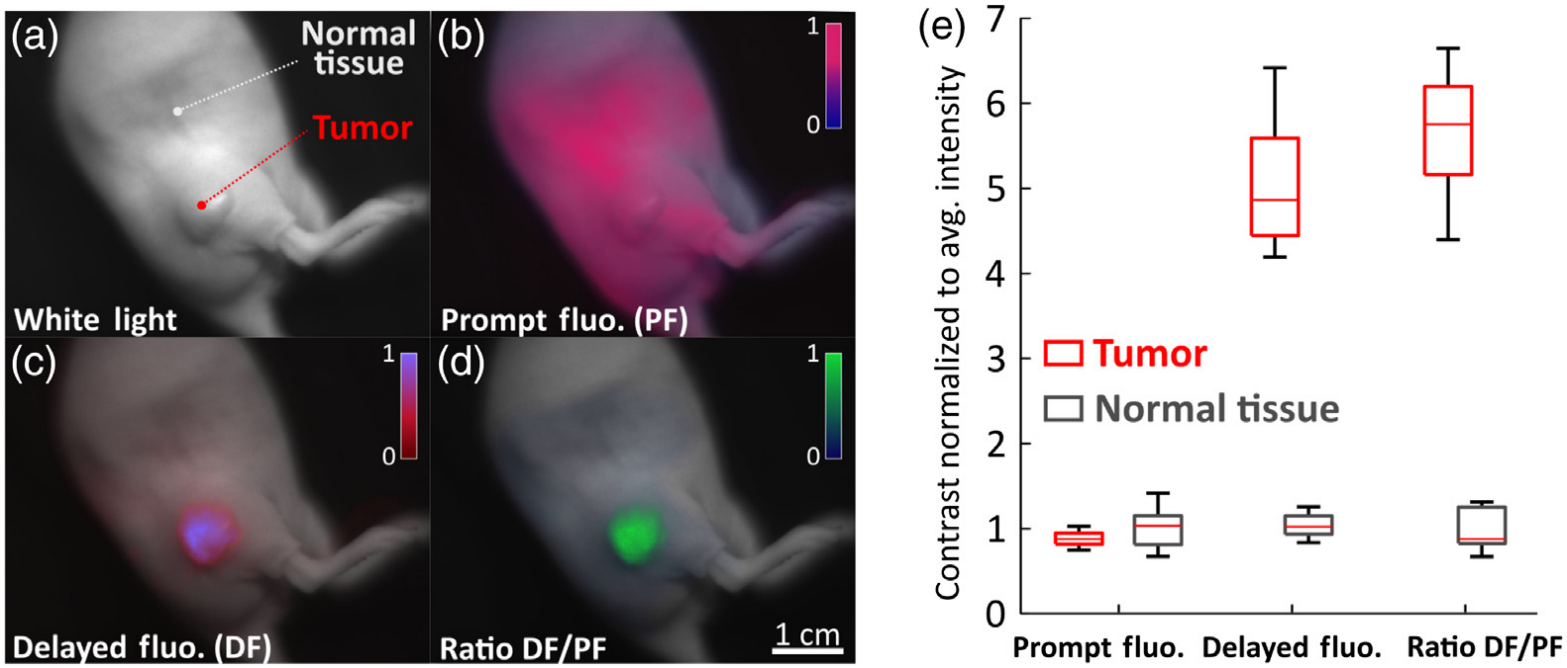
(a) White light image of a xenograft pancreatic tumor (BxPc3) on a mouse. (b) PpIX PF image of the mouse 6 h after i.p. administration of ALA. (c) PpIX DF image showing strong signal at the tumor because of its hypoxia. (d) DF to PF ratio allows for slightly improved contrast and corrects the output for PpIX concentration and tissue absorption. (e) Quantification of tumor visualization reliability using intensity values normalized to the maximum intensity for PF, DF, and R. ROIs were selected across the picture as shown in panel (a) (Video 1, mp4, 8.31 MB [URL: https://doi.org/10.1117/1.JBO.27.10.106005.s1])).

The PpIX distribution in tissues is dynamic and changes over time, with accumulation in tumors largely through the EPR effect, and high production in normal mouse skin. The PF signal typically has a higher signal in mouse skin than in tumors because of this dynamic. Imaging of a mouse to illustrate the whole skin-wide PF production is shown in Fig. 4(a) with PF in Fig. 4(b). In this case, it is apparent that the PF background signal is high [Fig. 4(b)]. Considering DF instead, there is a drastically increased contrast [Fig. 4(c)] in the tumor relative to the normal tissue at the same time point. Even though raw DF data shows outstanding contrast, considering the ratio DF/PF allows for slightly improved contrast and allows one to correct for PpIX concentration and tissue coloration [Fig. 4(d)]. It can be noted that the ratio R map is more homogenous across the tumor than the DF signal map. To quantify contrast, intensities normalized to normal tissue average intensities for each image are shown in Fig. 4(e) [with regions of interest shown in Fig. 4(a)]. While PF shows a similar signal in healthy and normal tissue, DF and R provide strong contrast. Although DF is related to the oxygen pressure in tissue, Fig. 4(c) shows a scale ranging from 0 to 1 because the system is not calibrated for absolute ${\mathrm{pO}}_{2}$ measurement. It is however not crucial for surgical guidance applications where mainly contrast between tumor and normal tissue is the key issue that matters.

### 3.5 Contrast Variation and Tissue Heterogeneities

**Fig. 5:**
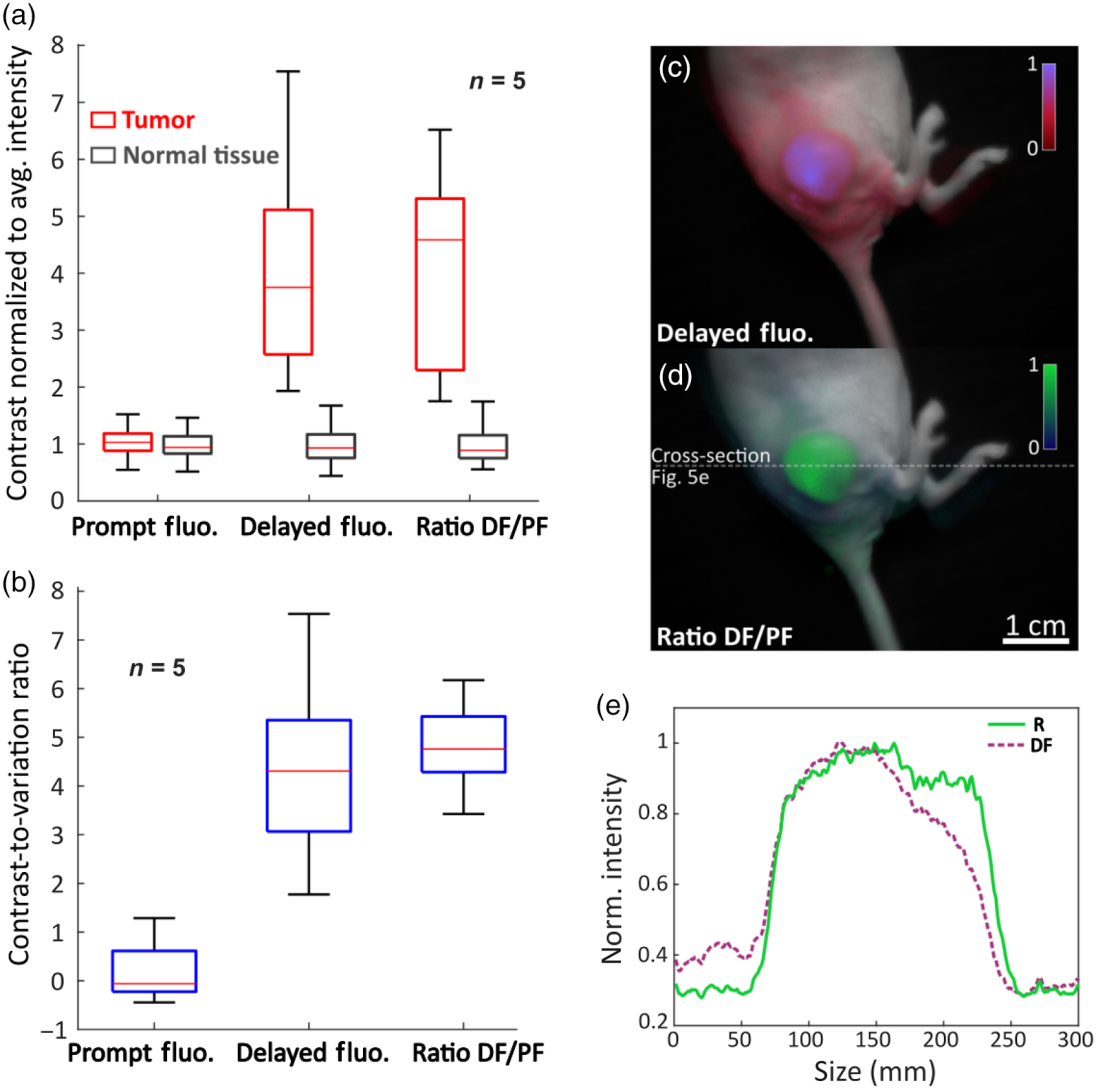
(a) Quantification of contrast between tumor and normal tissue for the three signals PF, DF, and the ratio R=DF/PF, for n=5. For each specimen, 10 ROIs were chosen for both the tumor and the normal tissues. (b) Shows for the same data, the CVR, considering heterogeneities in oxygen distribution. (c) and (d) Example of a specimen where R gave better contrast and finest edge distinction than DF. (e) Plot of the cross-section of DF and R [delimited by the gray dotted line on (d)] showing sharper edges distinction and better contrast in R.

Contrast quantifies the ability to distinguish differences in intensities between two objects. It is often difficult to define since it depends on many different factors. When imaging metabolism, like ${\mathrm{pO}}_{2}$ in tissue, it is particularly true since oxygen distributions can be extremely heterogenous, especially in tumors.^21^ Figure 5(a) summarizes data acquired from five mice, about 1 min after palpation. For each mouse, 10 regions of interest (ROI) were chosen over both the tumors and the normal tissues. PF signal showed no contrast, whereas DF and R showed varying contrast ranging from ∼2 to ∼7. This relatively large range is mostly due to oxygen heterogeneities giving a large range of readings for different ROIs and specimens. Extended exposition to anesthesia reduces heartbeat and leads to a decrease of the body temperature, causing vasoconstriction. Both these phenomena enhance hypoxia, particularly in the skin, leading to reduced contrast in certain cases. However, as shown on Figs. 5(c) and 5(d), visual contrast remains good. To account for these heterogeneities, CVR can be considered instead [Fig. 5(b)]. This ratio shows that both DF and R provide great contrast compared to PF, and that R provides better overall contrast. Another important factor regarding contrast for fluorescence-guided surgery is how well the tumor edges can be distinguished. Figure 5(e) shows that tumor edges definition is greatly improved in the ratio map R. This is because R accounts for PpIX concentration and differences in tissue coloration. Due to the animals breathing and the transients implied by palpation, contrast is a dynamic variable rather than a static value, depending on the specimen and its conditions, as well as on the tumor size and structure. The ratio R=DF/PF offers overall better contrast and improved edge differentiation than considering raw DF signal.

## 4 Discussion

While surgical oncology guidance is usually limited by the availability of optical tracers, ALA is a well-tolerated, FDA-approved drug, inducing excess intracellular PpIX production in most tissues. From this perspective, the ALA-PpIX system provides one of the most well-tolerated and commonly accepted methods to induce an optical metabolic contrast within tissue. It has seen broad applicability, but the imaging of the concentration alone provides the most robust contrast in a limited number of applications where there is inherent transport/delivery contrast with ALA or where there is low background in the surrounding normal tissues of PpIX production. However, what is presented here is a fundamentally different contrast mechanism, which is independent of the PpIX production or concentration and rather uses it as a reporter of tissue hypoxia.

Since PpIX interacts via collisional quenching of the triplet state with surrounding molecular oxygen, the reverse intersystem crossing has a very low probability and is essentially unseen in normal environments. However, the absence of oxygen leads to a much higher yield of this back to the excited singlet state of the molecule, and a subsequent fluorescence that has the lifetime of the triplet state decay, in hundreds of microseconds. This signal is easily measured with a fast time-gated camera coupled with a fast short-pulsed excitation and appropriate filtering. Thus, PpIX exhibits a DF with intensity inversely related to the tissue local oxygen partial pressure. Since it is produced as a precursor of hemoglobin, it is naturally synthesized by cells mitochondria and is therefore a unique tool to measure intracellular and extracellular oxygen *in vivo*. In this work, we developed an optimized method to gather both PF and DF signals in real-time, combining single photon sensitive technology and modulated acquisition timing.

This study was motivated by the need for surgeons to visualize oncologic tissue with specific contrast agents that are readily available, robust, and biologically reasonable. The discovery of this imaging method based on the tissue metabolism is a very good way to localize tumor tissues since they are known to be hypoxic, unlike almost all normal tissue. In the last decade, surgical guidance using ALA-induced PpIX has emerged but has been suffering from a lack of contrast between normal and tumor tissue because much of the focus is simply on the PF PpIX signal, which just reports on the produced concentration. Acquiring DF is a technological challenge but provides supplemental metabolic information that is linked to, but largely independent of, the heme synthesis cycle that produces PpIX concentration.

Hypoxia imaging based on PpIX DF intensity currently provides a qualitative reading of oxygen distribution in living tissue. Future work will focus on calibrating the DF response to obtain absolute measurements of ${\mathrm{pO}}_{2}$. One of the main challenges in achieving this task is to correlate PpIX ratiometric readings with another oxygen measuring standard. To date, PpIX DF is the only way to measure intracellular oxygen *in vivo* in a noninvasive way. The use of supplementary oxygen-sensitive optical probes such as PdG4^12^ could be an alternative for calibration.

Because PpIX DF intensity relies mostly on how much PpIX is present in the imaged tissue, tissue thickness or underlying organs can bias the fluorescent signal. Even though correction based on PF provides a coarse rectification, it cannot take into account ${\mathrm{pO}}_{2}$ variations across tissue depth. Using shorter excitation wavelengths with shorter penetration ranges would likely help with making the fluorescent signal less sensitive to tissue thickness and underlying organs.

Two particular biological features of PpIX were exploited here as well, and their realization is as important as the use of the DF signal. First, PpIX can be produced anywhere in the body and is diffused out and relocated around the body by the bloodstream. This feature is critically important because it means that most cancer tumors accumulate PpIX over time through the simple EPR effect that is the origin of the delivery of all drugs to cancer tumors. So, the method here does not rely upon the unique production of PpIX in the tumor itself, as it can come from anywhere in the body and relocate there. This feature is well known^22^ and provides the ideal way to achieve PpIX signals from most cancer tumors.

The second major biological feature examined here was that palpation can induce a transient hypoxia in tissue and that the kinetics of recovery of that are very different in tumors versus normal tissue. Again, this is a phenomenon that is very well known but not widely utilized as a contrast or imaging tool. Here, the transient hypoxia differences seen in these pancreatic cancers relative to the surrounding normal skin are quite striking and exceptionally high contrast. Palpation is a normal part of many cancer surgeries, as the surgeon determines which tissue feels harder or softer, as a contrast mechanism for resection. The combined use of palpation with hypoxia imaging may be a fundamentally new way to give the surgeon a visual que into the biology of the tissue they are looking at.

A major advantage of the presented technology is its compatibility with existing ALA PpIX surgery clinical workflow, which can enable a straightforward clinical translation. The main challenges to overcome are background light pollution that can interfere with the low DF signal and photobleaching of PpIX. When PpIX is exposed to light, it can degrade to photoproducts with different fluorescent properties. These photoproducts have similar excitation spectra and can exhibit DF with a shorter lifetime. However, it was determined that photobleaching only implies a consequent effect on DF measurement at irradiances $>10\;J/{\mathrm{cm}}^{2}$.^23^ With the current setup, this would translate to ∼6  h of continuous use of the hypoxia imager. In clinical settings, ALA is administered several hours before tumor resection (in the case of a brain tumor). PpIX can be imaged in the tumor as long as it is still present. We expect the time window for imaging DF of PpIX to be the same as the one used in the clinic. Since room lights are usually dimmed during this kind of procedure, photobleaching is not a problem and DF can be imaged over a few hours.

This pilot study aims to communicate this new technique out to other interested research groups. Further evaluation of the correlation between tumor margins, palpation, and the acquired signal across a wide range of preclinical and clinical cases, as well as the study of corresponding histopathological data, will be needed to assess the robustness of this imaging modality.

## 5 Conclusion

Hypoxia imaging for surgical guidance has never been possible, but here it is shown that PpIX has a unique emission in hypoxic tumor tissue regions, which is measured as a DF signal in the red to near-infrared spectrum. PpIX is an endogenous molecule approved by the FDA. Because most tumors have microregional hypoxia present, imaging hypoxia signals from PpIX DF allows for excellent contrast between normal tissue and tumors. In this work, we demonstrate >5× contrast in a pancreatic cancer model, relative to surrounding normal oxygenated tissues. Additionally, tissue palpation amplifies the signal and provides intuitive temporal contrast. The simple technology required, and the fast frame rate capability coupled with the low toxicity of PpIX make this mechanism for contrast translatable to human and could easily be used in the future as an intrinsic contrast mechanism for oncologic surgical guidance.

Hypoxia imaging for surgical guidance has never been possible, but here it was shown that PpIX has a unique emission in hypoxic tumor tissue regions, which can be imaged by a camera as DF signal in the red to near-infrared spectrum. PpIX is an endogenous molecule approved by the FDA. Because most tumors have microregional hypoxia present, imaging hypoxia signals from PpIX DF allows for excellent contrast between normal tissue and tumors. In this work, we demonstrate >5× contrast in a pancreatic cancer model, relative to surrounding normal oxygenated tissues. Additionally, tissue palpation amplifies the signal and provides intuitive temporal contrast. The key technology required, including a fast frame rate capability camera, coupled with the use of low toxicity of ALA to induce endogenous PpIX, are combed to make this mechanism for contrast. The concept is directly translatable to humans use and could easily be used in the future as an intrinsic contrast mechanism for oncologic surgical guidance.

## Code Availability

MATLAB routines for this study are available from the corresponding author upon request.
